# CARs-DB: A Database of Cryptic Amyloidogenic Regions in Intrinsically Disordered Proteins

**DOI:** 10.3389/fmolb.2022.882160

**Published:** 2022-05-18

**Authors:** Carlos Pintado-Grima, Oriol Bárcenas, Zoe Manglano-Artuñedo, Rita Vilaça, Sandra Macedo-Ribeiro, Irantzu Pallarès, Jaime Santos, Salvador Ventura

**Affiliations:** ^1^ Institut de Biotecnologia i de Biomedicina and Departament de Bioquímica i Biologia Molecular, Universitat Autònoma de Barcelona, Barcelona, Spain; ^2^ Instituto de Biologia Molecular e Celular and Instituto de Investigação e Inovação Em Saúde, Universidade Do Porto, Porto, Portugal

**Keywords:** amyloid, intrinsically disordered proteins, database, disease, protein-protein interactions

## Abstract

Proteome-wide analyses suggest that most globular proteins contain at least one amyloidogenic region, whereas these aggregation-prone segments are thought to be underrepresented in intrinsically disordered proteins (IDPs). In recent work, we reported that intrinsically disordered regions (IDRs) indeed sustain a significant amyloid load in the form of cryptic amyloidogenic regions (CARs). CARs are widespread in IDRs, but they are necessarily exposed to solvent, and thus they should be more polar and have a milder aggregation potential than conventional amyloid regions protected inside globular proteins. CARs are connected with IDPs function and, in particular, with the establishment of protein-protein interactions through their IDRs. However, their presence also appears associated with pathologies like cancer or Alzheimer’s disease. Given the relevance of CARs for both IDPs function and malfunction, we developed CARs-DB, a database containing precomputed predictions for all CARs present in the IDPs deposited in the DisProt database. This web tool allows for the fast and comprehensive exploration of previously unnoticed amyloidogenic regions embedded within IDRs sequences and might turn helpful in identifying disordered interacting regions. It contains >8,900 unique CARs identified in a total of 1711 IDRs. CARs-DB is freely available for users and can be accessed at http://carsdb.ppmclab.com. To validate CARs-DB, we demonstrate that two previously undescribed CARs selected from the database display full amyloidogenic potential. Overall, CARs-DB allows easy access to a previously unexplored amyloid sequence space.

## 1 Introduction

The information required for proteins to adopt their final conformation is naturally encoded in their amino acid sequence (Dill and MacCallum, 2012). Through evolution, optimal protein structures have been selected to perform diverse biological functions while avoiding the population of non-native conformations (Kuhlman and Baker, 2000). However, globular proteins cannot escape from bearing amyloidogenic sequences that might trigger undesired aggregation reactions, which are connected with an increasing number of human degenerative diseases (Invernizzi et al., 2012; Chiti and Dobson, 2017). These aggregation-prone regions (APRs) are usually buried in the hydrophobic core of the native state (Rousseau et al., 2006), and their presence responds to the thermodynamic and evolutionary coupling between the native and the amyloid states of globular proteins (Langenberg et al., 2020). In this context, the presence of APRs in Intrinsically Disordered Proteins (IDPs) has been traditionally considered marginal. The lack of a hydrophobic core and their biased composition towards polar residues is thought to confer them inherent protection against aggregation (Uversky et al., 2000).

Many globular protein functions involve the establishment of functional protein-protein interactions (PPIs), with the regions responsible for these contacts displaying significant amyloid potential, relative to the rest of the surface (Castillo and Ventura, 2009; Pechmann et al., 2009), reflecting the overlap in the requirements to set up functional and aberrant protein interactions (Pastore and Temussi, 2012). The high dynamism of IDPs allows for the interaction with several molecular partners (Dunker et al., 2005; Wright and Dyson, 2015; Arbesu et al., 2018), but the duality between function and aggregation was assumed not to apply for them.

Different bioinformatics tools have been designed to detect amyloidogenic regions from the primary sequence (Fernandez-Escamilla et al., 2004; Conchillo-Sole et al., 2007; Garbuzynskiy et al., 2010; Maurer-Stroh et al., 2010; Sormanni et al., 2015; Santos et al., 2020). Still, in their default configurations, the algorithms behind these methods identify amyloid regions that map at sequence segments of high hydrophobicity and aggregation propensity, that in the native state are often protected from the solvent at the core or interfaces. This has precluded the identification of protein sequences consisting of more hydrophilic and less aliphatic residues but still able to assemble autonomously and efficiently into amyloid fibrils (Diaz-Caballero et al., 2018; Hughes et al., 2018; Louros et al., 2020), which we have named cryptic amyloidogenic regions (CARs).

In recent work (Santos et al., 2021), we explored the prevalence of CARs of polar nature in IDPs by adapting the thresholding system of Waltz (Maurer-Stroh et al., 2010). This well-validated algorithm employs an empirical position-specific scoring matrix derived from the analysis of a database of short amyloid-forming and non-forming sequences. Our results indicated that CARs are widespread in IDPs, with 56–84% of IDRs exhibiting at least one region displaying amyloid propensity, depending on the applied threshold. The capacity of these regions to form amyloid fibrils was empirically tested for two short peptides from the human p53 and the retinoblastoma-associated protein. The analysis demonstrated that IDPs contain abundant mild amyloidogenic regions, which are preferentially involved in establishing functional PPIs, which were previously underestimated because of their polar nature. The data indicated that the price to pay for the presence of functional CARs is a concomitant risk for protein malfunction and disease, underpinning that the duality between functional and aberrant interactions also applies for IDPs. This possibility and the underrepresentation of validated amyloidogenic regions described for IDPs, compared to those in globular proteins, led us to build up CARs-DB, a database that collects related information for all detected CARs in DisProt, the most comprehensive database of experimentally validated IDPs (Quaglia et al., 2021).

The release of the CARs-DB database allows for a fast and straightforward search of thousands of predicted CARs without the need to perform time- and cost-demanding individual analyses. Also, the statistically significant linkage between CARs and IDPs interacting segments, especially when they follow a folding-upon-binding mechanism, makes CARs-DB an orthogonal tool to study these regions. Alternatively, the CARs-DB can be of help to identify regions at risk of promoting aberrant interactions that so far have remained masked. The amyloidogenic potential of CARs in the database is experimentally validated for two new short segments belonging to IDRs in titin and RPLP2 proteins.

## 2 Methods

### 2.1 Dataset Generation

To gather the data to generate the database, the sequences of all IDRs were downloaded in FASTA format from DisProt (2021_08 release), a manually curated database of disordered proteins (Quaglia et al., 2021). The Waltz algorithm was used to identify potential amyloidogenic segments. The “Best overall performance” of Waltz sets the detection threshold at a value of 92.0 (Maurer-Stroh et al., 2010), which performs very well on classic amyloid regions but is too high to identify CARs. We previously showed that thresholds of 85.0, 80.0 and 73.5 allowed us to identify and rank those regions according to their differential polar and ionizable residues content (Santos et al., 2021). IDRs longer than 20 amino acids were scanned with these parameters. Only CARs longer than six residues were included in the final database. Information about each detected CAR can be accessed through different links that map to its correspondent disordered region (DisProt) or the entire protein (UniProt).

### 2.2 Database Implementation

The server site was developed using the Django 3.0 web framework. The front-end is written in HTML/CSS. The database search engine is based on a JavaScript filter table that allows to retrieve CARs specific information upon querying by distinct identifiers. It is platform-independent and does not require previous registration.

### 2.3 Peptide Preparation and Aggregation

The peptides with the sequences Ac-EGVSISVYR-NH_2_ and Ac-GAVAVSAA-NH_2_ corresponding to the CARs-derived segment of titin and RPLP2 proteins respectively were purchased from Genscript Biotech (New Jersey, United States) with a purity >95%. Peptides were N-terminal and C-terminal protected as a strategy to mimic the protein environment. Peptide stock solutions were prepared by solubilizing the lyophilized peptides at a final concentration of 1 mg/ml in 100% dimethyl sulfoxide, divided into aliquots and stored at −80°C. For aggregation assays, peptides were diluted to 200 µM in phosphate saline buffer (PBS) and incubated at 37°C for 72h-7 days in a 96-well plate (SIGMA-Aldrich, Saint Louis, United States) under continuous agitation (100 rpm). The final concentration of DMSO in the assay was 4 and 1.3% (v/v) for titin and RPLP2 peptides, respectively. Additional details from the aggregation experiments are described on the MIRRAGGE spreadsheet (Martins et al., 2020) of the Supplementary material.

### 2.4 Binding to Amyloid Dyes

The fluorescence emission spectra of the binding of 40 µM Thioflavin-T (ThT) to the aggregated peptides were recorded using a Spark plate reader (Tecan, Männedorf, Switzerland). The samples were excited at 440 nm with an excitation bandwidth of 5 nm. The emission spectra were recorded from 460 to 600 nm with an emission bandwidth of 5 nm and a 2 nm interval.

Congo red (CR) interaction with the peptide solutions was tested using a Cary 100 UV/Vis Spectrophotometer (Varian, Palo Alto, United States). The absorbance spectra were recorded from 400 to 650 nm using a 1 cm optical length quartz cuvette at room temperature. 10 µl of peptide samples were preincubated with 90 µl of CR at a final concentration of 5 µM for 10 min at room temperature. Solutions of PBS with 5 µM CR and without peptide were used as negative controls.

### 2.5 Attenuated Total Reflectance Fourier Transform Infrared Spectroscopy

The secondary structure of incubated peptides was analyzed by attenuated total reflectance Fourier transform infrared (ATR FT-IR) spectroscopy using a Bruker Tensor FT-IR Spectrometer (Bruker, Massachusetts, United States) with a Golden Gate MKII ATR accessory. The aggregated peptide solutions were dried out under N_2_ (g) atmosphere. Each spectrum consisted of 32 scans and was measured at a spectral resolution of 4 cm^−1^ within the 1800–1,500 cm^−1^ range. All spectral data were acquired and normalized using the OPUS MIR Tensor 27 software and the Peak Fit 4.12 program (Systat Software Inc., San Jose, United States) was used for data deconvolution.

### 2.6 Transmission Electron Microscopy

For negative staining, aggregated peptide samples were sonicated at intensity two for 5 min in an ultrasonic bath (VWR ultrasonic cleaner, Leuven, Belgium), placed onto carbon-coated copper grids, and incubated for 1 min. The excess of the sample was removed using ashless filter paper. Then, the grids were washed with distilled water and incubated for 1 min with 2% (w/v) uranyl acetate and the excess of uranyl acetate was removed carefully using ashless filter paper. The micrographs of aggregated peptides were obtained using a TEM JEM-1400 (JEOL, Tokio, Japan) operating at an accelerating voltage of 120 kV. Representative images of each sample were selected.

## 3 Results

### 3.1 The CARs-DB Database, A Repository of Polar Amyloidogenic Peptides

#### 3.1.1 Web Interface

**FIGURE 1:**
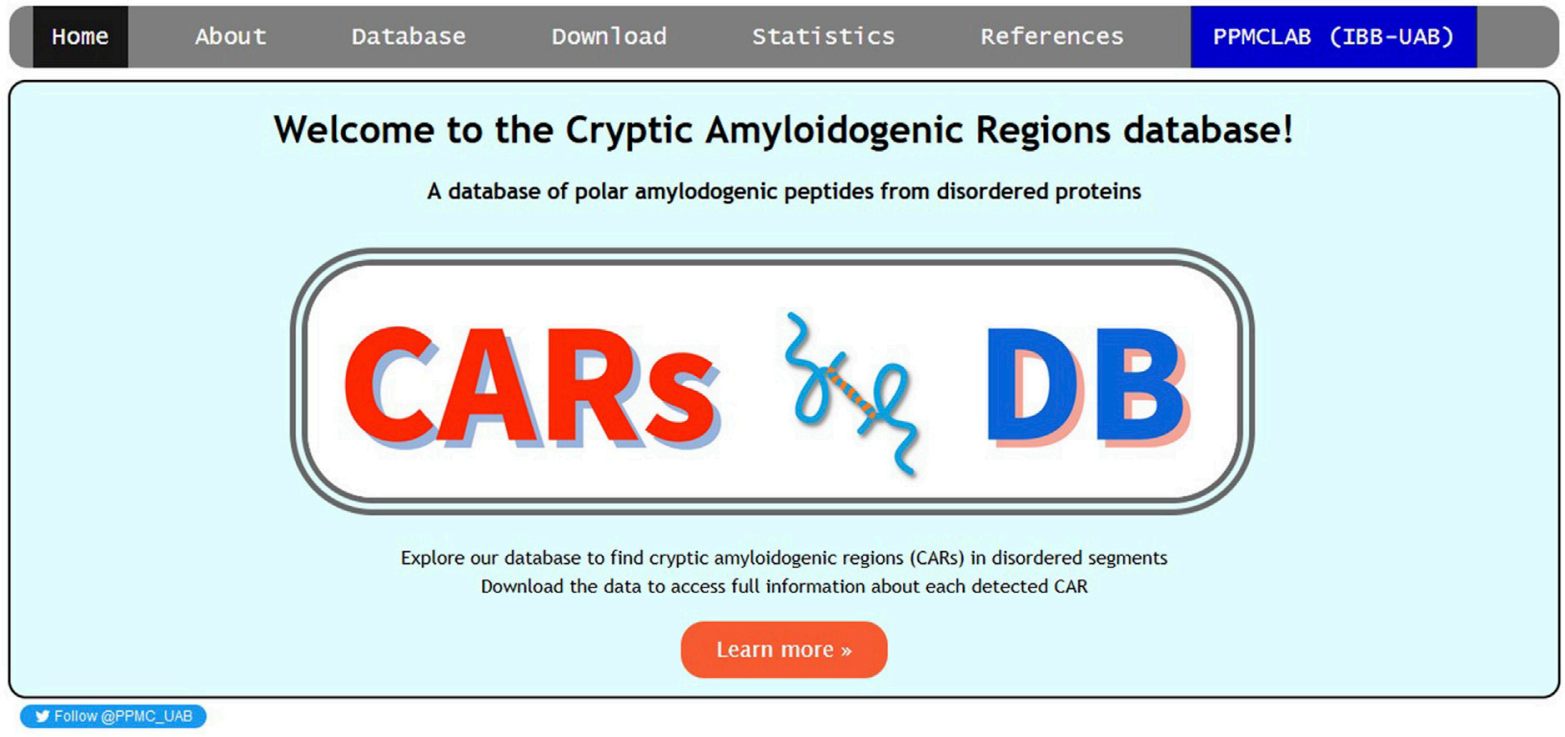
Screenshot of the homepage of the CARs-DB database. Different sections can be accessed to explore the features and content of the database through the navigation bar on the top of the site.

The CARs-DB is available at http://carsdb.ppmclab.com/. The server allows navigation through the different sections of the site. The main page welcomes the user and briefly describes the database’s general functions and content (Figure 1). In the ‘About’ section, a complete description of the research’s background and the documentation relative to the database is provided. If further help is needed, the source publications can be accessed under the ‘Reference’ section. We also included a ‘Statistics’ page to illustrate the abundance and distribution of CARs in the database and the nature of the amino acids that compose them.

#### 3.1.2 Database Content

The ‘Database’ section of the server gives access to a list of amyloidogenic regions detected at the three designated Waltz thresholds (85.0, 80.0, and 73.5), as described in the methods section. This allows for the generation of a comprehensive collection of diverse sequence stretches grouped according to their relative intrinsic amyloid propensity. Each CAR is individually associated with a unique DisProt ID and UniProt ID in this collection.

**FIGURE 2:**
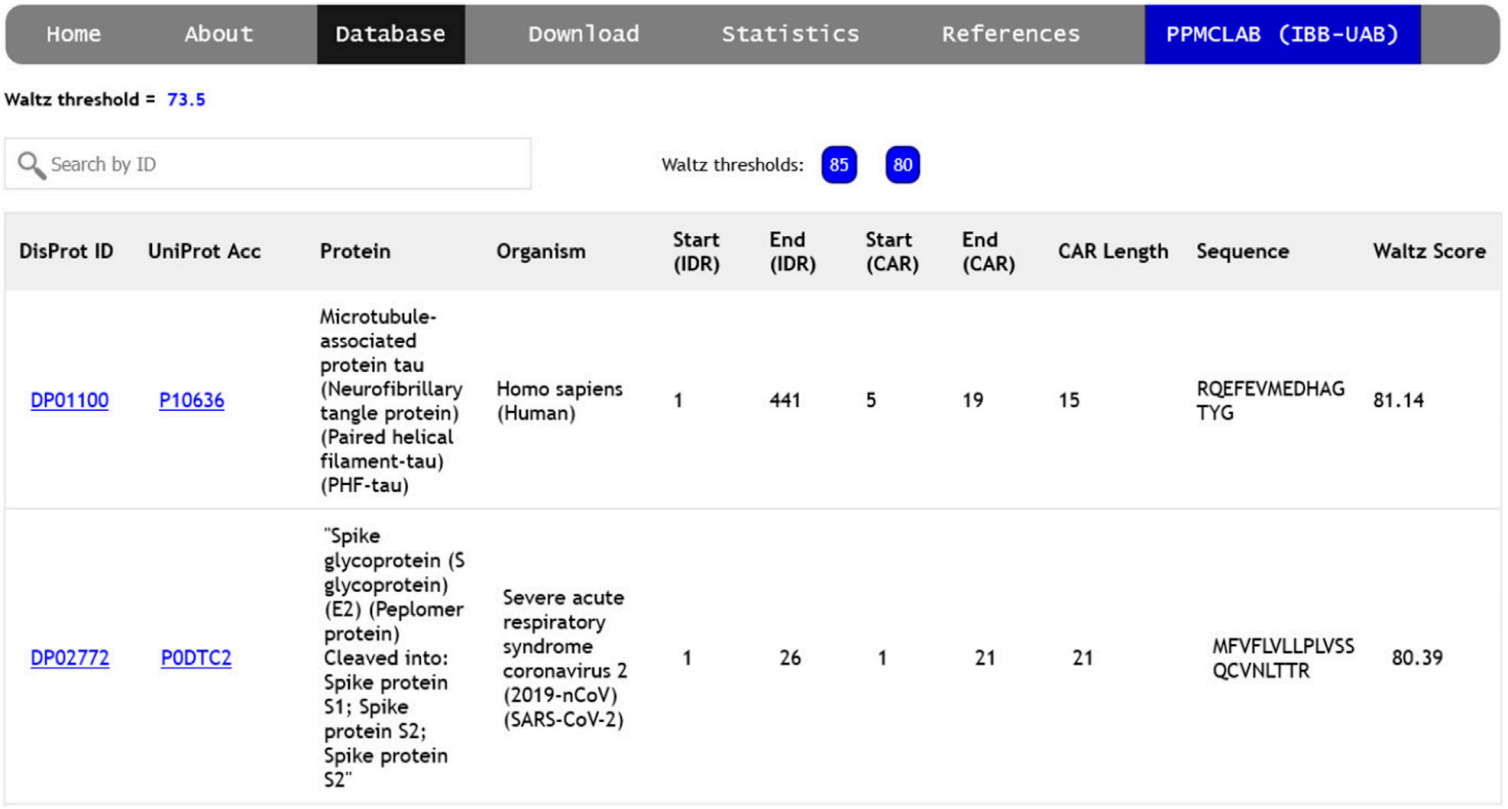
Screenshot of the ‘Database’ page. The different thresholds can be explored using the ‘85’, ‘80’ and ‘73.5’ buttons found next to the search bar. Each CAR is a new entry with its associated DisProt ID, UniProt code and protein name, source organism, disorder position and the results for the amyloidogenic prediction (CAR position, length, sequence and score).

The database can be accessed independently for each threshold, collecting relevant information for every CAR (Figure 2), including links to complete descriptions of the disordered region, the protein itself, and the source organism. The starting and ending positions of each CAR-containing IDR are indicated, relative to the entire protein sequence. CARs are defined by the first and last position of the identified peptide, their length, and amino acid sequence. Finally, the overall Waltz score for each prediction is shown in the rightmost column. Users can search the database by DisProt ID, UniProt accession number, protein name, source organism, or peptide sequence.

**FIGURE 3:**
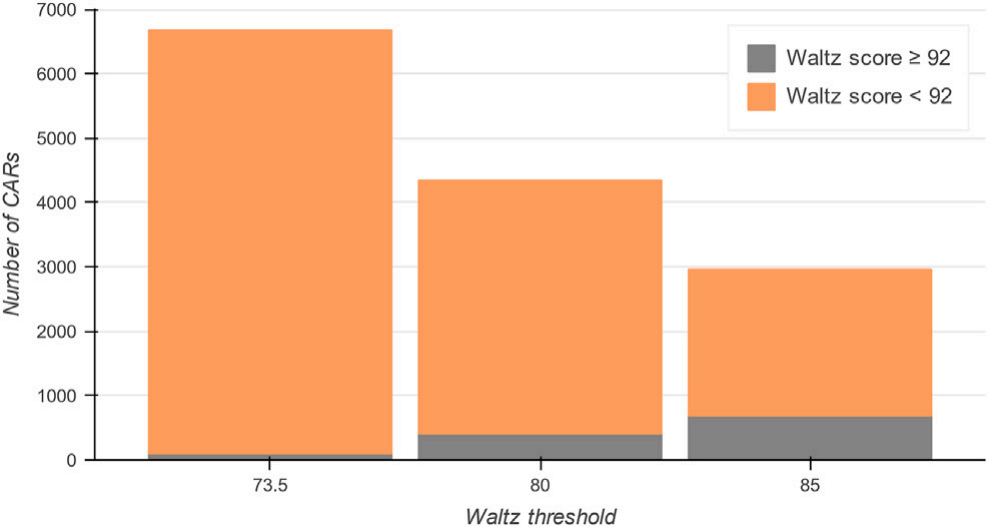
Total number of entries in the CARs-DB database for all DisProt regions. In grey, conventional amyloidogenic peptides are shown with a Waltz score over 92. The proportion of CARs dramatically increases as the threshold is more permissive (in orange), obtaining a collection of more than 8,900 unique CARs of polar nature.

To guide users through the content and organization of the database, a complete picture of the data within the CARs-DB can be found under the ‘Statistics’ section of the server. It includes graphical representations of the physicochemical properties of CARs and the database’s general content. The CARs-DB database contains a total of 2,962, 4,347, and 6,681 entries for the 85.0, 80.0, and 73.5 thresholds, respectively (Figure 3). Remarkably, a maximum of 686 regions with a Waltz score over 92.0, which is considered the threshold for bona fide conventional amyloids, were detected. This explains why when using the default parameters trained to identify these sequences, IDRs seem devoid of APRs. The statistics show that this insensitivity to CARs can be explained in terms of composition, as most of them present a polar nature with the presence of ionizable residues that are often depleted in conventional amyloidogenic regions. Overall, the analysis suggests that the amyloid sequence space might be significantly larger than what we traditionally considered and sequences with mild amyloid potential might be ubiquitous in IDRs.

The distribution of CARs by organism and threshold is reported at the ‘Statistics’ section of the database and in Supplementary Table S1, which details most of the relevant model organisms. *Homo sapiens* is the species with more associated entries, followed by *Saccharomyces cerevisiae*. Other relevant groups include viruses and prokaryotes other than *Escherichia coli*, with close to 10 and 8% representation in each case. The rest of the species fall under 6% representation.

#### 3.1.3 Data Availability

The content of the database is available under the ‘Download’ section of the server. The users can download the complete information in the desired file format to work locally. Three different file formats (CSV, TSV, and JSON) are provided for each threshold. Aside from all CAR-specific features, DisProt details for each IDR are also collected. These include the experimental evidence code (ECO), the IDP ontology term (IDPO), PubMed ID (PMID), and the position of the disordered region (start, end, and length).

### 3.2 Disease-Associated Regions Can Be Found With the CARs-DB

We believe that the CARs-DB can help identify regions at risk of promoting aberrant interactions that may eventually lead to pathology, since the presence of CARs has been associated with neurodegenerative diseases and cancer (Santos et al., 2021).

Amyloid formation has been experimentally validated for short peptides or motifs belonging to different IDPs linked to disease. That is the case of α-synuclein, an IDP whose deposition in dopaminergic neurons is intimately ligated to the onset of Parkinson’s disease (Luk et al., 2012). A stretch of 12 amino acids in the NAC domain of α-synuclein is essential for filament assembly (Giasson et al., 2001). We sought this short region (71-VTGVTAVAQKTV-82) in our CARs-DB and found it under the 73.5 threshold with a Waltz score of 78.51. Another interesting example is the microtubule-associated protein tau, an IDP that is found as intracellular solid aggregates in Alzheimer’s disease. A short hexapeptide in the C-terminal end of the protein (304-VQIVYK-312) is essential and sufficient for tau fibrillation (von Bergen et al., 2000; Meng et al., 2012). A CAR containing this peptide is present in the database with a Waltz score of 89.37. Aberrant aggregation can also disturb liquid-liquid phase separation (de Oliveira et al., 2019). In amyotrophic lateral sclerosis (ALS), the amyloidogenic core region of TDP-43 (residues 318–343) initiates its aggregation and facilitates its cytoplasmatic inclusion (Jiang et al., 2013). This region is positive at the 73.5 threshold and could not be detected with the default Waltz cutoff.

The amyloidogenic capacity of CARs in cancer-related proteins has also been demonstrated for peptides in p53 (326-EYFTLQIR-333) and retinoblastoma-associated protein (831-ILVSIGESFG-840) (Santos et al., 2021), both present in CARs-DB under the 85 and 80 thresholds, respectively.

Overall, these examples illustrate how amyloidogenic regions in pathogenic proteins may escape detection with conventional algorithms and the need to redefine the properties of amyloidogenic sequences since they can exhibit a marked polar character when they are embedded in a disordered context, as the regions listed in CARs-DB.

### 3.3 Orthogonal Identification of IDRs Involved in PPIs With CARs-DB

The dynamic nature of IDPs makes them highly exposed to the solvent and sensitive to the environment (Uversky, 2009). From an evolutionary point of view, the prevalence of exposed CARs in IDPs makes sense only if they contribute to the protein function since they necessarily endorse IDRs with a moderate but significant propensity to misassemble and malfunction (Santos et al., 2021).

The IDPs’ ability to establish interactions depends largely on the presence of modular interaction units in their sequences. These interaction-favoring regions are known as short linear motifs or linear interacting peptides (LIPs) (Davey et al., 2012; Pancsa and Fuxreiter, 2012; Piovesan et al., 2021). We demonstrated previously that the probability of any CAR residue to map into a LIP is > 2-fold higher than the one expected by chance (Santos et al., 2021), which suggested a role of CARs in facilitating IDPs’ PPIs. This is especially true when the interaction is mediated by a folding-upon-binding mechanism because the acquisition of structure in the bound state requires physicochemical properties that make the sequence more sensitive to aggregation in the unbound conformation (Langenberg et al., 2020).

The above observations suggest that CARs-DB might be used, in combination with dedicated applications, like ANCHOR (Dosztanyi et al., 2009), to detect LIPs susceptible to go on disorder-to-order transitions upon binding. To illustrate this potential, we describe four examples of CARs involved in disorder-to-order transition. These include short sequences from the receptor-binding domain (RBD) of the SARS-CoV-2 spike protein (482- GVEGFNCYFPLQSYGFQPTNGV-503, Waltz score = 80.1), the human microtubule-associated protein tau (350-VQSKIGSLDNITH-362, Waltz score = 82.0), the SUMO-interacting motif (SIM) PIAS2 (466-KVDVIDLTIESSSDEE-481, Waltz score = 87.1) and the p27^Kip1^ (p27) protein (70-LEGKYEWQEVEK-81, Waltz score = 75.4).

**FIGURE 4:**
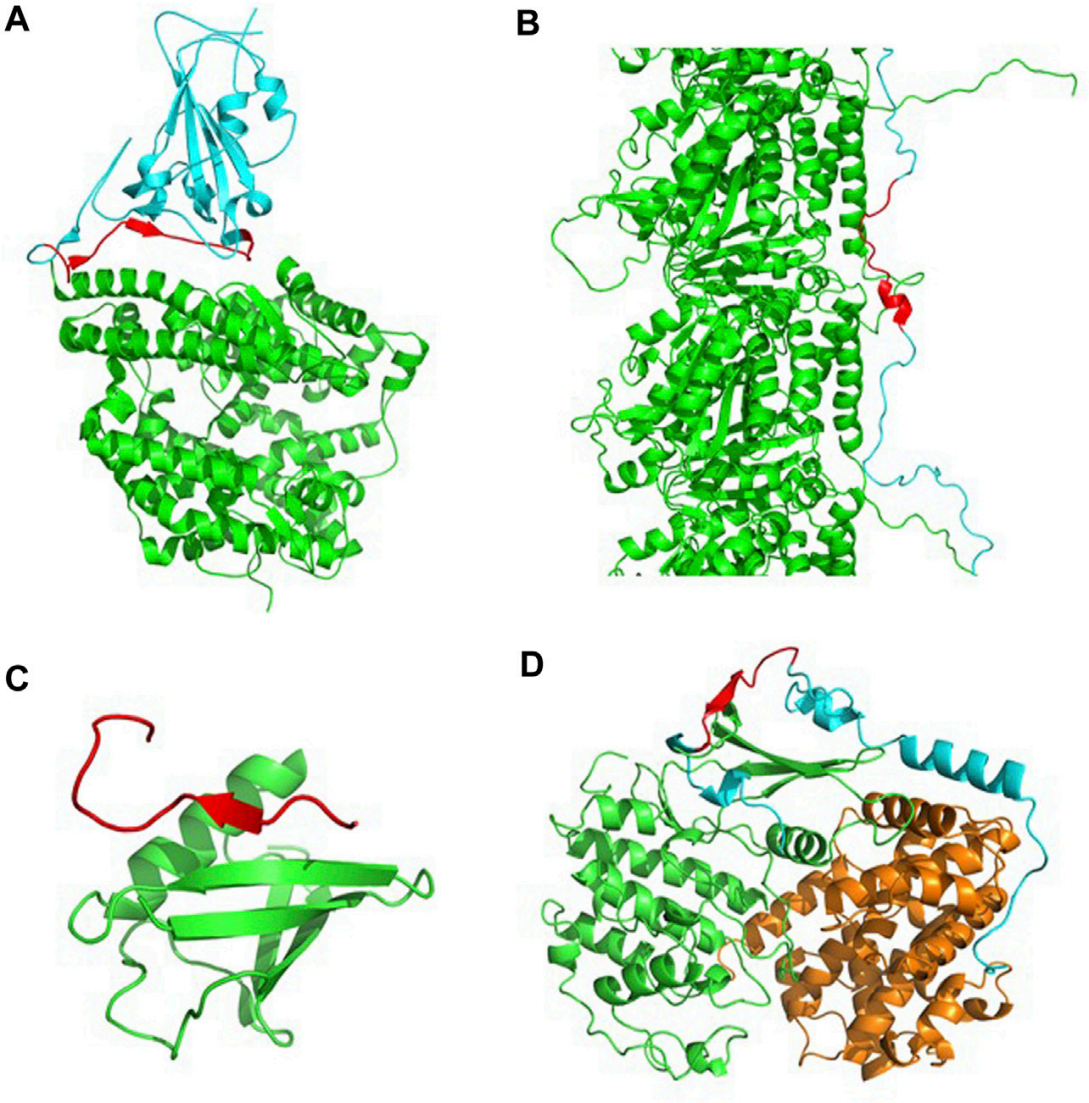
Identification of LIPs mediating PPIs selected from the CARs-DB. **(A)** The RBD of the SARS-CoV-2 spike protein (in cyan) in complex with its ACE2 receptor (in green) (PDB: 6LZG). In the interface, a 22-aa long CAR (in red) is found as a LIP that facilitates the recognition and entrance of the virus into the cell. **(B)** A segment of the tau-F isoform (in cyan) associated with tubulin-alpha-1B chain (in green) (PDB: 7PQP). A short CAR of 13 residues (in red) with an alpha-helix mediates functional interactions in the structural ensemble. **(C)** SIM-PIAS2 in complex with SUMO-1 (in green) (PDB: 2ASQ). A CAR of 16 residues with a local beta-sheet (in red) forms a SUMO-interaction motif that overlaps at functional interaction sites. **(D)** The p27 protein (in cyan) in complex with cyclin-dependent kinase 2 (in green) and cyclin A (in orange) (PDB: 1JSU). A polar CAR of 12 residues (in red) is found within an IDR that folds upon binding.

The RBD of the SARS-CoV-2 spike protein interacts with the angiotensin-converting enzyme-2 (ACE2) receptor found in human cells and is key to establishing cell host-virus interactions (Sternberg and Naujokat, 2020). Five antiparallel β-sheets form the core of this domain. From this core, it protrudes the receptor-binding motif (RBM), a region that is a flexible and disordered loop in the unbound state, and becomes ordered in the ACE2-bound SARS-CoV-2 Spike structure (Lan et al., 2020), while containing most of the interacting residues with the receptor. Interestingly, a 22-aa long CAR is found in the RBM overlapping with the RBD β6 strand in the bound state, indicative of an active role in binding (Figure 4A).

Tau is intrinsically disordered in solution and locally folds upon binding to microtubules (Kadavath et al., 2015). Specific intermolecular interactions stabilize the tau-microtubule complex (Brotzakis et al., 2021). Importantly, a Tau CAR of 12 residues is found to fold into an α-helical CAR within a strongly interacting Tau region that establishes contact with two α-helices of α-tubulin (Figure 4B). When the tau protein is detached from these structures, it becomes more sensitive to aggregation and may lead to the formation of neurofibrillary tangles, a trademark of the development of Alzheimer’s disease (Hardy and Selkoe, 2002).

Even protein families that are characterized by their high solubility might eventually self-assemble into β-sheet enriched aggregates (Sabate et al., 2012). It is the case of the ubiquitin-like SUMO proteins, which expose an aggregation-prone β-sheet needed for their binding to target proteins through their SUMO Interacting Motifs (SIMs). SIMs are disordered when unbound and form an intermolecular β-sheet with SUMO in the bound state. In the case of the PIAS2 SUMO substrate, its SIM fairly coincides with a 16 residues CAR (Figure 4C).

A canonical example of disorder-to-order transitions is p27, an IDP that folds upon binding to its Cdk/cyclin partners (Sivakolundu et al., 2005). The interaction regions encompass a 12 residues CAR that in the complex folds to form a short intermolecular β-sheet with the Cdk2 domain (Figure 4D).

Overall, in the four cases, the disordered nature of CARs turns into a partially folded structure upon binding to form the protein complex. This illustrates how CARs-DB may help to uncover LIPs displaying structural transitions upon PPIs which would have remained unnoticed with most conventional aggregation prediction approaches.

### 3.4 Experimental Validation of Novel CARs Forming Amyloid Fibrils

Providing additional experimental evidence on the amyloid potential of CARs would allow validation of the concept and justify the development of the CARs-DB. With this aim, here we tested the amyloidogenicity of two identified CARs involving the segment 10429-EGVSISVYR-10437 (Waltz score = 85.9) of titin protein (Q8WZ42) and the stretch 69-GAVAVSAA-76 (Waltz score = 75.3) region of S60 acidic ribosomal protein P2 (RPLP2) (P05387). None of the two proteins have been previously demonstrated to contain amyloid-forming regions.

Titin, the largest known polypeptide, constitutes the elastic filament of the sarcomere, playing a pivotal role in passive muscle force. The modular organization of titin comprises four main regions: the N-terminal domain, the I-band and A-band, and the C-terminal domain (Eldemire et al., 2021). Titin’s elastic capacity is mainly attributed to the I-band, and more specifically to the PEVK segment, an intrinsically disordered region where ∼75% of the amino acids are either proline (P), glutamate (E), valine (V), or lysine (K) (Nagy et al., 2005; Sudarshi Premawardhana et al., 2020). Nevertheless, the molecular interactions that contribute to PEVK’s elastic nature remain poorly understood. We predicted a 9-residue CAR comprising residues 10,429 to 10,437, belonging to the 9,880 to 12,031 disordered sequence that maps at the PEVK region.

As a second candidate, we selected RPLP2, an acidic phosphoprotein that is part of the ribosomal stalk and acts during translation elongation (Campos et al., 2020). The C-terminal domain of RPLP2 is intrinsically disordered and highly conserved as it is determinant in the recruitment of the factor eEF2 (Mishra et al., 2014; Campos et al., 2020), which is essential in the translocation step during translation elongation. We selected an 8-residue CAR corresponding to residues 69–76 mapping at the IDR comprising residues 63–115 of the C-terminal domain.

**FIGURE 5:**
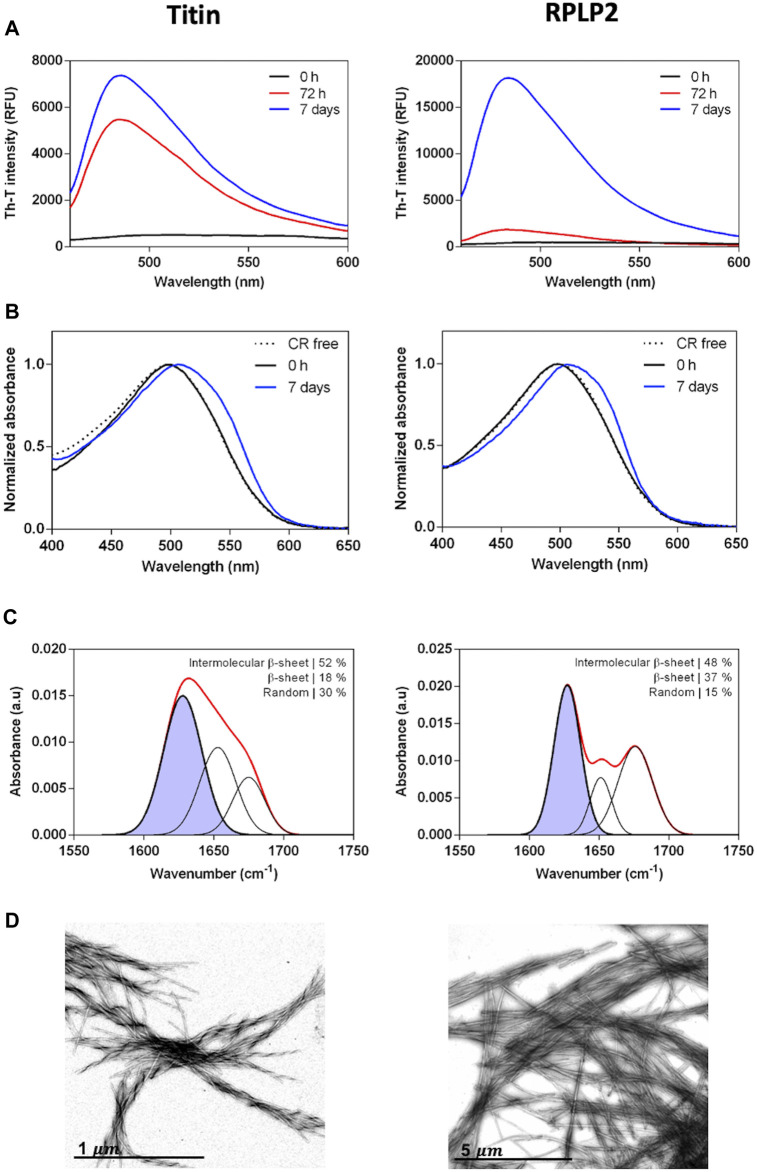
Experimental characterization of the selected CARs-DB derived peptides, the 10429-EGVSISVYR-10437 segment of titin protein (left panels) and 69-GAVAVSAA-76 of RPLP2 (right panels). **(A)** Thioflavin-T fluorescence emission spectra at 0 h, 72 h and 7 days of 200 µM of the peptides incubated at 37°C with continuous agitation at 100 rpm. **(B)** CR absorbance spectra of the incubated samples at 0 h and 7 days. CR absorbance spectrum in the absence of peptide is included as a control (dashed line). **(C)** Determination of the secondary structure of incubated peptides by ATR FT-IR. The red line corresponds to the absorbance spectra and the purple area indicates the inter-molecular β-sheet contribution to the total area upon Gaussian deconvolution. **(D)** Representative TEM micrographs of the peptide fibrils.

We assessed the *in vitro* self-assembly properties of the two selected CARs. The peptides were prepared at 200 µM in PBS and incubated at 37°C and 100 rpm for 72 h or 7 days. First, aggregation into amyloid-like structures from its initial soluble state was evaluated using the amyloid-specific dyes Thioflavin-T (Th-T) and Congo Red (CR). Both peptides promoted a high increase in the Th-T fluorescence emission that evolves significantly with time, reaching a maximum after 7 days (Figure 5A). Accordingly, subsequent experiments were all performed at 7 days of incubation time. As expected, the incubated peptides also displayed increased CR absorbance and the characteristic amyloid redshift of the spectra maximum compared to the free dye (Figure 5B). These results suggest that these peptides have the potential to aggregate into amyloid-like structures. Next, to inspect the secondary structure content of the assemblies, we used FT-IR spectroscopy and recorded the amide I region of the spectra (1,700–1,600 cm^−1^), corresponding to the absorption of the carbonyl bond group of the protein backbone. The deconvolution of the spectra allowed us to identify the main peaks in the 1,620–1,630 cm^−1^ region, accounting for approximately 50% of the total area in both peptides, indicating that they have acquired a predominant intermolecular β-sheet structure (Figure 5C). Finally, we performed morphological analysis using transmission electron microscopy to confirm peptides’ internal fibrillar structure. In agreement with the previous results, both solutions contained canonical amyloid fibrils that are twisted in the titin peptide, whereas in the PRLP2 peptide, they are straight and tend to associate laterally (Figure 5D).

Together, the above-described experimental data provide compelling evidence for the amyloidogenicity of these CARs-DB derived peptides.

## 4 Discussion

It is becoming increasingly clear that the physicochemical properties of regions underlying fibril assembly may vary considerably, ranging from the highly hydrophobic, β-sheet prone sequences that form the core of thermodynamically stable globular proteins to stretches of more polar and disordered nature present in IDPs (Sabate et al., 2012; Yoon et al., 2014; Marinelli et al., 2018; Louros et al., 2020; Santos et al., 2021). Currently, the large majority of reported amyloidogenic sequences belong to the first group, whereas representants of the second class are still scarce. The CARs-DB database we present here represents an effort to correct this unbalance, providing a repertoire of sequences bearing an amyloid potential compatible with their exposure to solvent in flexible IDRs, as demonstrated here experimentally for CARs belonging to the IDRs of two unrelated proteins, titin, and PRLP2.

The CARs-DB database consists of a precalculated dataset of more than 8,900 unique CARs from all experimentally characterized IDRs described in the DisProt database (Quaglia et al., 2021). This sequence source should allow researchers to rapidly identify non-classical amyloid-forming sequences within IDPs that would otherwise remain obscured by the intrinsic bias towards non-polar sequences of amyloid-detecting algorithms when used with their default detection thresholds. Besides, the database can be exploited as a complementary tool to study the potential structural transitions experimented by regions involved in IDPs PPIs, as shown here for CARs belonging to the IDRs of the SARS-CoV-2 spike protein, Tau, SIM-PIAS2 and p27 human proteins, or to spot critical regions that might lead to aberrant interactions associated to pathology.

In the context of the so-called amyloid origin of life (Greenwald and Riek, 2012; Greenwald et al., 2016; Maury, 2018), we have recently proposed that an early step in the evolution of proteins would have been the conversion of CARs-like sequences placed within short IDRs into interacting regions that allow for the formation of homotypic and heterotypic contacts (Santos et al., 2021). This mechanism could be at the origin of the first oligomeric complexes and coacervates, a substrate on top of which globular proteins might have appeared and evolved. CARs-DB provides access to a previously uncharted amyloid sequence space to validate or refute this intriguing but exciting hypothesis experimentally.

## Data Availability

The datasets generated for this study can be found under the ‘Download’ section of the database at http://carsdb.ppmclab.com/download. Available data is described in section 3.1.3 of this article.
