# Supplementary material for: CARs-DB: A Database of Cryptic Amyloidogenic Regions in Intrinsically Disordered Proteins
Source: Front Mol Biosci. 2022 May 18;9:882160. doi: 10.3389/fmolb.2022.882160 (PMC9309178; doi:10.3389/fmolb.2022.882160)
Supplement: Supplementary file 2 [file Table1.DOCX]

*Supplementary Material* for

CARs-DB: a database of Cryptic Amyloidogenic Regions in Intrinsically Disordered Proteins

Carlos Pintado-Grima, Oriol Bárcenas, Zoe Manglano-Artuñedo, Rita Vilaça, Sandra Macedo-Ribeiro, Irantzu Pallarès, Jaime Santos and Salvador Ventura*

**Supplementary Table 1.** Distribution of CARs by source organism and Waltz threshold. For each species and threshold, the number of CARs and its percentage is included. The proportion of entries by species remains unaltered in the different Waltz thresholds.

|  | 73.5 | | 80 | | 85 | |
| --- | --- | --- | --- | --- | --- | --- |
|  | Total | % | Total | % | Total | % |
| *H. sapiens* (Human) | 2467 | 36.92% | 1587 | 36.50% | 1090 | 36.79% |
| *M. musculus* (Mouse) | 391 | 5.85% | 247 | 5.68% | 165 | 5.57% |
| *R. norvegicus* (Rat) | 183 | 2.74% | 105 | 2.41% | 53 | 1.79% |
| *D. melanogaster* (Fruit fly) | 104 | 1.56% | 64 | 1.47% | 45 | 1.52% |
| *C. elegans* | 148 | 2.21% | 66 | 1.52% | 61 | 2.06% |
| *S. cerevisiae* (Baker's yeast) | 884 | 13.23% | 641 | 14.74% | 448 | 15.12% |
| *S. pombe* (Fission yeast) | 120 | 1.80% | 84 | 1.93% | 47 | 1.59% |
| *A. thaliana* (Mouse-ear cress) | 194 | 2.90% | 113 | 2.60% | 64 | 2.16% |
| *E. coli* | 218 | 3.26% | 138 | 3.17% | 87 | 2.94% |
| Other prokaryotes | 524 | 7.84% | 390 | 8.97% | 278 | 9.38% |
| Virus | 641 | 9.59% | 488 | 11.22% | 311 | 10.50% |
| Other | 808 | 12.09% | 425 | 9.77% | 314 | 10.60% |
| Total | 6681 | | 4347 | | 2962 | |
